# Agency via Awareness: A Unifying Meta-Process in Psychotherapy

**DOI:** 10.3389/fpsyg.2021.698655

**Published:** 2021-07-14

**Authors:** Eugenia I. Gorlin, Vera Békés

**Affiliations:** Ferkauf Graduate School of Psychology, Yeshiva University, New York, NY, United States

**Keywords:** psychotherapy, change process, common factor, awareness, agency

## Abstract

To address the need for conceptual and clinical consensus within the field, psychotherapy research has increasingly focused on identifying common principles of change. While the field contends that this approach is atheoretical, we argue that principles of change cannot be fully understood or applied without the context of some theoretical framework. This article develops such a framework by identifying and explicating two theoretical assumptions that are implicitly shared by multiple therapeutic approaches: (1) that increasing agency is a fundamental aim of psychotherapy, and (2) that therapists enhance clients' agency by increasing their awareness. Building on the largely disparate empirical literatures demonstrating the importance of client agency and awareness to successful therapeutic outcomes, we provide a theoretical account of the highly iterative and synergistic meta-process by which these two factors jointly produce change. Explicit identification and empirical investigation of this Agency via Awareness psychotherapy meta-process, we argue, could facilitate scientific and clinical progress within the field. The hypothesized meta-process is discussed in relation to existing integrative models of therapeutic change, and its manifestations in the theory and practice of major therapeutic orientations are reviewed and illustrated. We discuss how this framework can facilitate psychotherapy research by providing a common language and conceptual foundation for wide-ranging therapeutic approaches, constructs, and findings. Finally, by raising clinicians' awareness of the implicit assumptions underlying their therapeutic work, we suggest that the Agency via Awareness framework can increase *their* agency over when and how they apply these assumptions in therapy to maximize client improvement.

## Introduction

Much has been written about the pressing need to consolidate the accumulated scientific and clinical knowledge of psychotherapy, which currently remains scattered across disparate orientations that have largely talked past each other (e.g., Castonguay and Beutler, 2006; Goldfried, 2019). To address this need, the field has increasingly moved toward identifying and testing common therapeutic principles that lead to clinical improvement. These principles are designed to be less abstract than overarching theories (e.g., psychodynamic, behavioral, cognitive) but broader than specific techniques (e.g., interpretation, systematic desensitization, cognitive restructuring) (Castonguay and Beutler, 2006). As such, they are intended to provide a common understanding and language for the therapeutic processes that demonstrably lead to change, while sidestepping the seemingly irreconcilable theoretical disagreements that have kept the field divided. For example, Goldfried (1980, 2019) identifies five principles of change that are especially robust across treatments and have extensive scientific support: (1) “promoting client expectation and motivation that therapy can help”; (2) “establishing an optimal therapeutic alliance”; (3) “facilitating client awareness of the factors associated with his or her difficulties”; (4) “encouraging the client to engage in corrective experiences”; and (5) “emphasizing ongoing reality testing in the client's life” (Goldfried, 2019, p. 488).

These principles parsimoniously describe a wide range of therapeutic techniques and strategies that go by different names and are accompanied by different theoretical assumptions across orientations: for example, the principle of “fostering corrective experiences” is achieved through various therapeutic actions related to insight in psychodynamic therapy (Sharpless and Barber, 2012), “behavioral experiments” in cognitive therapy (Salkovskis, 1991), “exposure” in traditional behavioral therapies (De Silva and Rachman, 1981), “opposite action” (Linehan and Swenson, 2007) or “committed action” (Hayes S. C. et al., 2012) in “third-wave” therapies, and so on.

This identification of a limited number of scientifically sound and clinically useful change principles is a tremendous improvement over the proliferation of pre-packaged treatments that preceded them. By understanding some of the change processes that therapy aims to foster, a therapist gains greater flexibility and creative fluency in enacting whatever techniques will help foster those processes for a given client in a given situation, rather than rigidly implementing prescribed techniques that may not fit the client's current needs (e.g., Castonguay et al., 1996; Beitman et al., 2005). This is especially useful given recent studies showing that most therapists identify with several psychotherapy orientations simultaneously (e.g., Tasca et al., 2015; Békés and Aafjes-van Doorn, 2020). Common factor research has successfully identified intervention processes that cut across the majority of therapy approaches, which likely offers the benefit of building a common language, reducing friction, and thus allowing for more constructive conversations and idea exchanges between individuals allied with the different therapy schools.

As noted earlier, however, this approach is deliberately atheoretical, in the sense that it specifies processes for which there is empirical evidence, but does not posit any particular underlying theoretical assumptions that might explain and give rise to these processes. We submit that this is not a complete or permanent solution, because the principles of change cannot be fully understood or applied outside the context of *some* theoretical framework. As has been noted elsewhere (e.g., Madill and Doherty, 1994; Avdi, 2008), there is no truly atheoretical practice, given that any therapeutic choices a clinician makes will necessarily reflect certain implicit or explicit assumptions about the goals and causal mechanisms of therapeutic change. To decide, for example, what factors are “associated with [the client's] difficulties” (Goldfried, 2019, p. 488) and need to be brought to awareness, a clinician needs some account of what counts as a “problem”: for instance, by what and whose (e.g., client's, therapist's, society's) standard do we determine whether a given symptom or characteristic is problematic? Likewise, to determine what kinds of corrective experiences (Goldfried, 2019) a client needs and how to bring them about, the therapist needs to have some idea about the nature of what is to be corrected—a way of relating to others? A personality structure? A belief? A conditioned behavior?—as well as the causal mechanisms that may lead to change. Indeed, the nature and process of corrective experiences have been a topic of debate within and across therapy orientations (see Castonguay and Hill, 2012). Similarly, what counts as an “optimal” working alliance (Goldfried, 2019, p. 488) may depend on the nature of the work that the dyad intends to undertake: is it more akin to an alliance between two thought partners? Between two fellow humans navigating life's challenges? Between expert and consumer? Between mentor and student, or doctor and patient? Absent an explicit articulation of our underlying assumptions, we regress back to the problem we started with: a lot of competing models of how and when to apply these principles, with no common conceptual foundation to guide such decisions. Moreover, the identified common change principles do not specify causal relationships between mechanisms of change and the effects of clinical procedures, which would be necessary for any clinical theory (Hoffart and Hoffart, 2014). There is no escaping the need to engage with theory; the question is only how openly and critically we engage with it.

This paper thus aims to identify a set of core theoretical assumptions that are at least implicitly shared by a wide range of therapeutic modalities and existing meta-theoretical models of therapeutic change. Making these assumptions explicit affords several advantages: first, it makes these assumptions more transparent and subject to critical reflection and empirical testing; second, it provides a common conceptual foundation and deeper causal understanding for a wide range of previously disparate psychotherapy processes, techniques, and findings both within and across orientations; and finally, it makes previously identified therapy principles more clinically useful by providing a framework for selecting and applying them. The hypothesized core assumptions are: (1) that *increasing the client's agency over her life is a fundamental goal of psychotherapy*, and (2) that *agency is increased primarily by increasing the client's awareness of aspects of internal and external reality relevant to living her life*. The therapeutic upshot of these assumptions is that “therapy fosters agency by increasing awareness.” Below, we elaborate on these assumptions by defining and explaining our conceptualization of their key terms, review the ways they manifest in and inform the implementation of different therapeutic modalities, and discuss how the explicit adoption of these assumptions could aid theoretical and clinical progress within our field.

## Key Terms Explained

### Defining and Conceptualizing “Agency”

Bandura (2006) defined “an agent” as one who “influence[s] intentionally one's functioning and life circumstances” (p. 164). Accordingly, we conceptualize a therapy client's degree of “agency” in terms of the extent to which they demonstrate a capacity to choose and execute their own intentions in various domains of their life—such as their mental and emotional health, their work and relationships, the home they inhabit, or the communities in which they belong. In line with prior accounts of mental and physical action as distinct and interactive constituents of human agency (e.g., Mele, 1997; Mackrill, 2009; Metzinger, 2017; Pezzulo, 2018), we distinguish between *mental agency* (i.e., the intentional management of one's own internal mental processes, such as what one attends to, what memories or observations or questions one chooses to reflect on, etc.) and physical agency (i.e., the intentional management of one's external environment through physical action). Of note, physical agency as defined here presupposes some degree of mental agency, given that a client cannot form and enact intentions in the physical world without some degree of agency over the intentions they form, and over when and how they attend to those intentions and translate them into action plans (e.g., Achtziger and Gollwitzer, 2018).

Even if a client is fully agential, as we have conceptualized it here, this does not mean that they have limitless power over every situation, nor does it guarantee that all of their intentions will be successfully fulfilled. Rather, it means that they engage in the intentional mental and physical actions that constitute self-authorship and tend to generate success over time: e.g., they are able to attend to, reflect on, and, when needed, make peace with painful emotions, situations, and memories without reflexively reacting to them; to choose their own realistic and desirable goals and action strategies and take action in light of them; to acknowledge and learn from failures and setbacks, and flexibly adjust their intentions and/or strategies accordingly. In short, it means that they are an overall competent and responsible manager over the project of living their life.

#### Continuity With Prior Empirical Research

Therapists across psychotherapy traditions are in agreement that enhancing clients' agency in psychotherapy is a central aim of the psychotherapy process (Williams and Levitt, 2007), and both theoretical and empirical studies have supported agency's fundamental impact on optimal therapy outcomes (Mackrill, 2009). Various aspects of agency have been operationalized in terms of the client's contribution to the therapy process, openness, expressiveness, cooperativeness, autonomy, contribution to the therapeutic bond, active collaboration, and interactive collaboration with the therapist (Orlinsky et al., 2004). A recent meta-analyses showed that patient collaboration had a medium effect on therapy outcome (Tryon et al., 2019), and quality of patient participation in therapy has been presented as the single most important determinant of therapy outcome (see review by Orlinsky et al., 2004).

Furthermore, agency has been linked to stronger therapeutic alliance (Coleman and Neimeyer, 2015), and there is some research evidence showing that problems of agency might be related to treatment efficacy through therapeutic alliance, in that among patients with problems with interpersonal agency at the start of treatment, stronger alliance may lead to more improvement in depression symptoms over the course of therapy (Gómez Penedo et al., 2020). An integrative review by Ryan et al. (2011) identified patient autonomy as a common concern across wide-ranging psychotherapy and counseling approaches, despite being “differentially grounded in theories and differentially implemented in approaches” (p. 193). Presenting agency problems at the onset of therapy is common in patients (Toivonen et al., 2019, 2020), and thus regaining a sense of agency has been identified as a crucial focus in psychotherapy practice (e.g., Lilliengren and Werbart, 2010; Levitt et al., 2016; Wahlström and Seilonen, 2016). When analyzing patient narratives over the course of therapy, a stable increase in agency was found across patient characteristics (e.g., demographics, personality traits), and in various conditions (e.g., Kristmannsdottir et al., 2019). Increase in agency was indeed found to be related to improvement in mental health; moreover, increases in agency occurred prior to improvement in mental health (Adler, 2012). Consequently, recommendations have been made for therapists to facilitate developing or increasing a sense of agency in patients over the course of therapy (e.g., Williams and Levitt, 2007; Todd, 2014; von der Lippe et al., 2019).

A recently developed patient agency measure, the Therapeutic Agency Inventory (Huber et al., 2019), assesses agency specifically in the context of psychotherapy, that is, patients' intentional influence over the process of psychotherapeutic change. Therapeutic agency has been found to be related to lower psychological distress, lower depression scores, and better therapy outcomes even when controlling for baseline distress (Huber et al., 2019). Furthermore, positive changes in agency predicted subsequent symptom improvement in patients (*n* = 386) in psychodynamic outpatient psychotherapy (Huber et al., 2021).

These studies approach agency as a common factor in psychotherapy, and it may well be. However, we propose that promoting agency is a more fundamental, underlying aim of psychotherapy, which, together with the work of increasing awareness, constitutes an overarching therapeutic meta-process that underlies the efficacy of wide-ranging therapeutic approaches.

### Defining and Conceptualizing “Awareness”

By “awareness,” we are referring broadly to the grasp of whatever knowledge or understanding about oneself and the world is relevant to managing one's life. This conception of “awareness” encompasses both the “descriptive” and “interpretive” aspects of experience studied by phenomenological approaches (e.g., Matua and Van Der Wal, 2015). It includes, for example, an awareness of what kinds of careers or relationships or lifestyles are plausibly within one's reach, and what activities, experiences, risks, rewards, skills, and challenges would be involved in pursuing each one; how one feels and thinks toward each of these possibilities, and why; the nature and malleability of human emotions, beliefs, motivations, and habits, to inform one's understanding and predictions about how these will unfold in oneself and others over time; and, indeed, what steps one can take to in order to gain the needed awareness in the first place. One's awareness can increase through various mechanisms: in a therapeutic context, this may include encountering new knowledge for the first time (e.g., when a client learns about the physical symptoms of anxiety), bringing previously encountered knowledge back into consciousness (e.g., when a client recalls how it felt to be anxious as a child), making new connections that deepen or extend one's understanding of new and/or previously encountered knowledge (e.g., when a client realizes how their present-day anxiety relates to the anxiety they felt in childhood, or how anxiety has caused them to miss out on important opportunities), testing and internalizing new knowledge by applying it in action (e.g., when a client deliberately pursues an opportunity that they previously avoided due to anxiety), and consciously engaging with previously avoided knowledge (e.g., when a client acknowledges feeling anxious).

Therapy promotes increased awareness in various ways: for instance, by fostering *exploration* and drawing *attention* to new knowledge, and by facilitating *reflection* on and *application* of previously encountered knowledge. The more a client engages in these processes, in turn, the more deeply *internalized—*i.e., better elaborated, more integrated, and more readily and consistently accessible—their awareness becomes. As such, it gains increasing power to stir the client's emotions and motivate their actions. Fully internalized awareness, on this understanding, is akin to what some scholars have referred to as embodied cognition (e.g., Ignatow, 2007) or experiential knowledge (Given, 2008). In lay terms, such knowledge is experienced as having fully “sunk in,” or as “going all the way down.”

Thus, for instance, a client may report knowing “on an intellectual level” that “my drinking habit is a big problem,” but that does not yet mean she has an internalized, emotionally accessible awareness of the nature of the problem, or that this knowledge is “fully real” to her. For instance, if she has not consciously connected her drinking to the conflicts in her marriage, her dissatisfaction and underperformance at work, her children's emotional difficulties, or the painful ways she experienced her own parents' drinking when she was a child, then her awareness may be at the level of a vague speculation, unlikely to move her to work toward quitting or cutting down. Only by exploring, attending to, and reflecting on these connections can she make her awareness of the “problem” experientially vivid and emotionally salient enough that she is moved to apply it in physical action. This action, in turn, will allow her to create experiences that further deepen her awareness (e.g., increased efficacy at work, improved communication with her family) and, in turn, motivate further agential action toward solving the problems that led to and were caused by her drinking behavior.

Of note, internalized awareness does not necessarily need to be formulated explicitly. As numerous authors have pointed out, a large proportion of learning in therapy consists of implicit processes, such as by learning about new ways to relate to another person through the therapeutic relationship (Stern et al., 1998; Békés and Hoffman, 2020). For example, a client may gradually learn, through her work in therapy, that she is capable of loving and being loved, without ever putting this conviction into words. What makes this internalized awareness, rather than a mere belief, is that it rests on a foundation of relevant observations and corrective experiences (Castonguay and Hill, 2012) that have themselves been actively processed and systematically incorporated into her knowledge base, even if they were painful or hard to integrate (Stiles and Brinegar, 2007). As such, she can access the relevant particulars of her internalized knowledge and reliably bring them to bear on her subsequent action choices, even if she never articulates the underlying generalization. Of note, however, the further step of articulating their awareness explicitly does afford clients a further degree of agency, insofar as they are now able to reflect upon it critically and independently, understand its basis and origins, and consciously apply it in situations where it might not automatically occur to them. This also likely increases the likelihood that they will retain their newly internalized awareness even after therapy is done, as they are now able to rehearse and elaborate it in words (e.g., Sobczak and Gaskell, 2019).

#### Continuity With Prior Empirical Research

The general notion of gaining awareness during the course of psychotherapy has been studied under various terms, each approaching the process from different angles and emphasizing various aspects. Influential concepts and terms representing aspects of awareness include insight (Castonguay and Hill, 2007; McAleavey and Castonguay, 2014), knowledge, understanding, emotional processing (Kramer et al., 2015), corrective experience (Castonguay and Hill, 2012), ability to describe patterns, and affective awareness (Høglend and Hagtvet, 2019). These have been presented as important mechanisms of change across a range of therapy orientations (Wampold et al., 2007; Høglend and Hagtvet, 2019), such as psychodynamic therapy (Messer and McWilliams, 2007; Sharpless and Barber, 2012), experiential therapy (Pascual-Leone and Greenberg, 2007), and cognitive behavior therapy (Grosse Holtforth et al., 2007; Hayes A. M. et al., 2012), and in general, as core processes in psychotherapy (see reviews and meta-analysis by Connolly Gibbons et al., 2007; Jennissen et al., 2018). In addition, there is converging evidence that patients' increased awareness is linked to treatment outcome (Connolly Gibbons et al., 2009; Johansson et al., 2010; Kallestad et al., 2010; Kramer et al., 2015; Høglend and Hagtvet, 2019). As already noted above, the process of increasing awareness has been identified in prior research as a mechanism of therapeutic change across multiple treatment modalities (see Goldfried, 2019). More broadly, converging evidence from behavioral, neurobiological, and applied clinical research suggests that present-moment awareness of the affective consequences of one's current behavior is necessary for motivating sustainable behavior change (Ludwig et al., 2020).

## The Agency Via Awareness Framework

Colloquially, the essence of our proposed theoretical model is that “knowledge is power”: the wider and deeper our awareness of the reality in which we are living, the more freely and effectively we can operate within it. Just as importantly, we also contend that we have *power over our knowledge*, and that helping clients exercise this power is one of the fundamental tasks of therapy. Specifically, the mechanisms for increasing awareness (exploration, attention, reflection, and application) are all themselves agential processes. Exploring, attending, reflecting, and acting in pursuit of new knowledge requires effort and willingness, and thus is hard work (see Gorlin and Schuur, 2019). As such, we refer to the process of increasing awareness in psychotherapy as itself an *agential process*. The motivation to engage in this agential process must itself come from some degree of awareness that doing so is likely to be both tolerable and worthwhile. This awareness may be experienced as a sense of curiosity, or as an implicit recognition that one's problems have generally become more manageable the more one has come to understand them, or as a generalized sense of trust in the authenticity and personal relevance of the new knowledge one is encountering (akin to what Fonagy and colleagues have termed “epistemic trust”; Fonagy and Allison, 2014; Fonagy et al., 2019).

**Figure 1:**
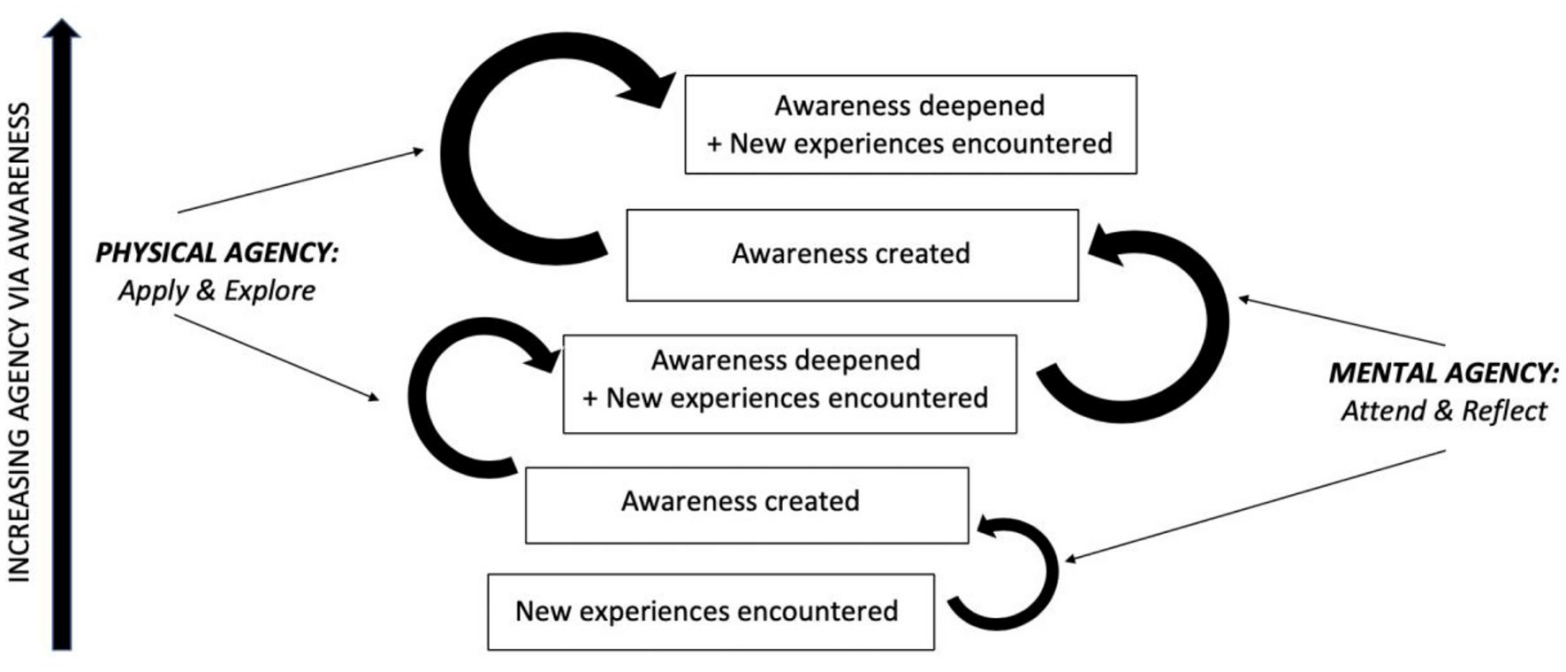
Schematic illustration of agency via awareness meta-process aimed at in psychotherapy. The rounded arrows, starting from the bottom, represent the successful exercise of mental and physical agency, whereas the text boxes represent stages of increasing awareness. As demonstrated in the figure, new awareness is created via attention to and reflection (mental agency) on new (internal or external, encountered or created) experiences; this awareness is then applied (to internal and/or external experiences) and inspires further exploration, which leads to gradual deepening of awareness as well as further new (internal or external, encountered or created) experiences, thus providing further opportunities for creating or deepening awareness and thereby increasing agency. Of note, clients may get “stuck” at any of these stages, and the aim of therapy is to provide the experiences and/or facilitate the agential work that will allow clients to make forward progress through this spiral.

By the same token, the very first steps of psychotherapy, in which the client seeks out help, contacts a therapist, and shows up at the appointment, already represent physically agential acts—perhaps the most agential acts available to the client at the moment. These acts, in turn, presuppose some degree of prior awareness that the client is experiencing significant problems and has not been able to solve these problems on their own, as well as some degree of mental agency in attending to and reflecting on the experiences that led to this awareness. This prior agential work and resulting increased awareness then give rise to the opportunity to gain further awareness during the therapy process, which, in turn, will inform further agential work in exploring and acting based on what is learned. Thus, agency and awareness are interconnected in a virtuous spiral (see Figure 1), with one leading to the increase of the other.

This dynamic interplay between agency and awareness is particularly crucial given that lack of awareness can result from a mix of primarily cognitive (e.g., not having encountered the information before, or having unintentionally forgotten or misinterpreted it) and primarily motivational factors (e.g., defensively rationalizing away or avoiding contact with the information because it is threatening, painful, or hard to integrate). To further complicate matters, these two factors routinely interact: for instance, if a client unintentionally misinterprets others' benign criticisms as signaling malicious or hostile intent, she will likely be more motivated to avoid discovering their true intent (e.g., by minimizing further contact with them or telling herself “I don't care what they think”), which in turn will cut her off from the very information that might serve to correct her initial misinterpretation. By conceptualizing such a case through the “Agency via Awareness” lens, a therapist would be equipped to recognize the role that both factors are playing, and to intervene accordingly. By asking “Where is this client stuck in the agency-via-awareness meta-process?,” the therapist could recognize that she lacks awareness of the benign intentions held by at least some of the people in her life, which currently motivates her to avoid fully attending to and reflecting on other people's intentions for her (i.e., exercising her mental agency), which in turn prevents her from gaining awareness of the rewards that could follow from opening herself up to more trusting and intimate relationships (i.e., exercising her physical agency). The therapist might thus intervene by providing a corrective experience that the client would be likely to attend to (e.g., by self-disclosing the therapist's own benign intentions toward her in a moment when she defensively withdraws). Alternatively, the therapist might choose to gently call the client's attention to prior instances when she derived value from attending more fully to previously avoided aspects of her experience, thus increasing her motivation to do so again in this instance. Which point of intervention the therapist chooses would depend on which of these aspects of the client's reality they can most readily help her access in a tangible form, which may vary based on the shared experiences between client and therapist to date, what intervention techniques the therapist feels most comfortable delivering, etc. What the therapist informed by our model likely would *not* do, however, is (1) take the client's statement that “I don't care what other people think” at face value and attempt to talk her out of it via cognitive restructuring, or (2) offer a detached, impersonal interpretation of the client's defensiveness without providing any means for her to reevaluate her assumption of others' hostility. Thus, our model provides a flexible but principled standard by which to tailor one's treatment to a given patient and therapeutic context, anchored to a theoretical understanding of what one is fundamentally trying to achieve.

Thus, agency and awareness often go hand-in-hand in the therapy process, as they are inextricably connected. Exploring, attending to, reflecting on, and pursuing new experiences is often itself effortful work requiring either (and more often both) mental or physical agency. Moreover, whereas agential work is typically depicted as inquisitive and action-oriented, in reality, willing acceptance and surrender to unchangeable aspects of internal and external reality (Safran, 2016) can be just as agential in our view. Indeed, for clients struggling to make sense of deep loss, failure, misfortune, or injustice, this can be extremely painful and thus courageous work. But it is work that may need doing if one is to envision and enact a more satisfying life. It is agential work. The fundamental task of therapy, we contend, is to inspire, empower, and support this work.

## Convergence With Existing Integrative Models of Therapeutic Change

The Agency via Awareness framework aligns with and provides a common conceptual foundation for multiple existing integrative models of the therapeutic change process. For example, Mackrill (2009) presents an Existentialist definition of client agency as encompassing the various ways in which clients intentionally affect the physical world, themselves, other people, and their lives as a whole, both in and out of therapy. Of note, he particularly highlights the role of reflection and self-understanding as crucial to the process of exercising agency over one's own agential functioning, in line with the role we ascribe to increasing awareness in our model. As Mackrill observes, psychotherapy researchers have conceptualized and operationalized client agency in many distinct ways, with each approach capturing some aspects of the overarching construct and neglecting or underemphasizing others. Of particular relevance to our identification of Agency via Awareness as a meta-process of therapeutic change, he identifies two theoretical models that operationalize agency in terms of “general client change processes”: Stiles et al. (1990) assimilation model and Prochaska's transtheoretical model of change (Prochaska and DiClemente, 1983; Prochaska and Norcross, 2002). Both of these models describe sequences of psychological stages and corresponding therapeutic processes that clients undergo in the course of adaptive change, with both sequences involving a clear progression from increased awareness to increased agency. For Stiles et al. (1990), the stages proceed from an initial grasping and emotional processing of a problematic experience that has been warded off from awareness, to the gradual puzzling out, understanding, problem-solving, and integrating of that experience into one's overall knowledge base, to the application of this newly integrated knowledge in action, culminating in a sense of mastery. For Prochaska et al., the stages progress from precontemplation to contemplation to preparation to action to maintenance: a clear progression from increasing awareness to agential action on its basis. The 10 processes posited to underlie these stages similarly progress from increasing awareness (e.g., via consciousness raising, environmental reevaluation, self-reevaluation, and dramatic relief) to informed actions (e.g., via self-liberation, counter-conditioning, helping relationships, and stimulus control). Importantly, although the model's stages and processes are outlined sequentially, Prochaska and Velicer (1997) emphasize that people will often move through these stages in an iterative, nonlinear fashion. Similarly, as described earlier and presented in Figure 1, we expect that clients routinely expand their awareness by applying it in action, both to keep previously acquired knowledge accessible in awareness (especially while it is still cementing) and to update and further internalize it in light of new experiences.

Analogously, Allen (2007) identifies “active agency” as one of three unifying elements across recent “metatheories” of therapeutic change (Mahoney, 2003; Magnavita, 2005; Scaturo, 2005). Although he does not explicitly identify “awareness” as a unifying element in these frameworks, his account of “active agency” presupposes at least some degree of awareness about the choices one has available and the factors that inform those choices, as manifest, for example, in one's “rational problem-solving ability” (p. 277) and one's “insight into the origins of a problem,” which “help[s] patients and their significant others think about their problems in new ways” (p. 283). In discussing the other two unifying elements he identifies—a complex system of interacting levels” and “dynamic tension between stability vs. change and individuation vs. attachment”—he repeatedly invokes the role of new and internalized awareness in helping clients navigate this complexity and find their own resolution to these tensions.

Similarly, Goldfried (1980, 2019) five common principles of change, as described above, can readily be understood as stages in the overarching process of increasing agency via awareness. Since expanding their awareness is effortful, emotionally charged, and often courageous work for clients, it requires *motivation*, which in turn requires at least some initial awareness of the expected rewards and of the therapist's trustworthiness in guiding the patient toward those rewards (as reflected in the need for a strong *therapeutic alliance*). Then follow the principles of *gaining new awareness, engaging in corrective experiences*, and *ongoing reality-testing*: all inherent aspects of the agential work of increasing and internalizing awareness. The aim of all these processes is to help clients “behav[e] in ways that are more conducive to adaptive functioning” (Goldfried, 2019, p. 489); in other words, to exercise greater agency over their lives. Yet these theorized causal connections between the five processes, and the reciprocal Agency via Awareness meta-process that holds them together, are not made explicit in Goldfried's deliberately atheoretical model, nor in any of the other models described here. This theoretical account of the tight, iterative causal relationship between agency and awareness, such that they not only enable but are partly constitutive of one another, is what our proposed theoretical model contributes.

## Agency Via Awareness Across Therapeutic Modalities

In addition to the integrative models of change described above, a wide range of specific therapeutic approaches, principles, stages, and techniques can be understood and integrated through this Agency via Awareness conceptual lens. Each of these varied approaches, we contend, derives its value from its role in helping people explore, attend to, reflect on, and apply new knowledge about internal and external reality, so that they have more freedom and control over their choices and life in general. Notably, this is an implicit aim even of therapeutic modalities that explicitly disavow the possibility of awareness, agency, or both. A particularly salient example is behavioral therapy, which evolved out of an animal-based theoretical model that explicitly precluded awareness—or any internal mental state—from exerting a causal effect on behavior (Moore et al., 2008). As such, it also precluded clients' capacity for agential control over their behavior, as we have defined it: according to Skinner (1971), our notion of ourselves as initiating agents is a mere illusion, given that behavior is fully caused by chains of environmental contingencies. Yet, in the implementation of this theoretical framework with human adults, awareness and agency inevitably come to play significant roles, given it is the client who bears ultimate responsibility for managing their own environmental contingencies. Ryan et al. (2011) note this discrepancy in their account of behavioral therapy approaches:

“[Behavior therapy's] emphasis on clients' volition, voice, and input in the context of therapy does not appear to us to be particularly theory-derived. Nonetheless, this emphasis on clients' experience of choice and self-endorsement of treatment goals seems strongly emphasized in practice manuals and in our personal interactions with behavior therapists.” (p. 208)

Thus, for instance, most behavioral therapy manuals (e.g., Goldfried and Davison, 1994; Spiegler and Guevremont, 1998) emphasize the importance of explaining the treatment rationale, building clients' awareness of the environmental cues that trigger their problem behavior, and thereby empowering them to exercise agency over their own habitual responses (via techniques like exposure and stimulus control) so that unhelpful stimulus-response associations can gradually be unlearned. These observations align with Bandura et al. (1977) influential finding that the therapeutic effects of an ostensibly behavioral, performance-based intervention for phobia were entirely mediated by changes in self-efficacy (i.e., internal expectancies about one's ability to cope with one's feared situation). Building on this idea, recent theory and research from an inhibitory learning perspective (Craske et al., 2008) suggests that stimulus-response associations themselves represent internalized attitudes or expectancies that are formed through experience and can be checked and updated through the work of increasing awareness (see Craske et al., 2014). Likewise, operant conditioning exerts its effects on behavior via internalized causal expectancies about the positive or negative consequences of acting a certain way: e.g., a client might assume that “If I interact with strangers, I will be mocked and bullied.” Once the client becomes aware of this expectancy, she is better positioned to check it against her present-day reality, perhaps realizing that the conditions in which she formed this belief as a child are no longer applicable. This new awareness, in turn, gives her the agency to practice acting contrary to the fear that accompanies her internalized expectancy (e.g., via social cost exposures; Nelson et al., 2010), long enough to deepen and solidify her awareness that interacting with strangers is less dangerous and ultimately more rewarding than she previously believed. In this light, contingency management strategies (Hayes and Hofmann, 2018) can be conceptualized as awareness-prompting mechanisms. By planting associative reminders in the environment, clients ensure that they are able to bring the relevant knowledge into awareness when it is needed, without having that knowledge eclipsed by previously learned but no longer endorsed associations (e.g., the tendency to associate novel social situations with danger). For instance, if a client is undergoing social cost exposures to deepen the awareness that interacting with strangers is rewarding and safe, they may be able to bolster their agency by treating themselves to a tangible reward after every exposure, thus keeping the knowledge of its long-term value in their awareness.

Analogously, the concept of “psychic determinism” in Freud's psychoanalytic theory held that, in the psychological realm, all conscious choices are determined by unconscious forces; thus, there is no “agent” outside these forces who would be unaffected by them, or, put differently, there are multiple agents in the conscious and unconscious realms. As such, in his view, the existence of full agency as in absolute freedom to choose is merely an illusion and ultimately impossible (Freud, 1965, p. 236, id. Wooldridge, 2018). However, even though unconscious forces are thought to undermine the individual's agential aspirations, the aim of analysis is to become aware of these forces and integrate them into a more coherent sense of agency, thus increasing autonomy (Moran, 1993; Wooldridge, 2018). Psychodynamic therapies focus on self-reflection as a way to increase awareness of unconscious forces and processes. The overarching goal of these therapies, at least as they are practiced today, is to “expand freedom and choice by helping people to become more mindful of their experience in the here and now” (Shedler, 2010, p. 14). Depending on the particular approach, the emphasis may be on reflection on past events that might shape the patient's present experiences, on the patient's defense mechanisms, or on the therapy relationship as a vehicle for shedding light on the patient's general relational patterns (e.g., Fonagy and Bateman, 2006). Nearly all psychodynamic therapies, however, share the supposition that gaining insight over previously unconscious motivating forces is a central mechanism underlying symptom reduction and personal growth. By bringing unconscious material into consciousness, the patient becomes aware of their own, previously unconscious internal processes and motivating forces. This awareness, in turn, increases the patient's agency by allowing increased mastery over previously unconscious processes (per the famous quote: “Where id was there shall be ego”, Freud, 1965) and control over the drives that led to the development of symptoms and/or prevented the patient from acting freely, according to their will.

## Clinical Application

Clients may present in therapy with a wide range of agential deficits, all of which, we contend, are accompanied by corresponding deficits in awareness. For instance, some clients may exhibit domain-general impairments in mental agency that manifest in an inability to form distinct, stable attitudes or intentions independent of the therapist or some other external authority (Toivonen et al., 2019). In more overt cases, this may take the form of distressing identity confusion and a manifestly unstable sense of self, as is regarded to be a defining feature of borderline personality disorder (Gold and Kyratsous, 2017). Of note, this general lack of mental agency is typically accompanied by a lack of clear, differentiated awareness of one's own emotions, motivations, and thought processes, which naturally restricts one's ability to form and maintain differentiated attitudes and intentions in light of them.

Other clients may exhibit relatively robust, independently formed attitudes and intentions but struggle to muster the motivation or sustain the focus required to act on them consistently (as is often the case for those with major depression and attention deficit problems, respectively). With respect to awareness, such clients may exhibit a lack of practical knowledge about aspects of the world that would be relevant to enacting their intentions (e.g., they may mention wanting to “work with computers” but have somewhat vague or unrealistic expectations about the skills or qualifications required) and/or about the nature of the psychological obstacles they are encountering (e.g., they attribute their attentional difficulties to “laziness” or believe that depression is a permanent and untreatable condition).

Still others may experience more local, domain-specific difficulties in forming decisive intentions within a given realm (like one's career, family, or romantic life), reconciling certain internal conflicts (between a desire for intimacy and a fear of rejection, for example), or overcoming certain mental or physical roadblocks (such as crippling anxiety, a behavioral addiction, or a medical illness). These problems, in turn, are typically accompanied by either lacking or insufficiently internalized awareness or specific misconceptions about the relevant domain(s) (e.g., not being fully aware of their own feelings in or own ways of contributing to a certain relationship dynamic, not realizing there are more like-minded people in the world than those they have met to date, or not having sufficiently internalized the knowledge that anxiety tends to become more rather than less manageable once we face the objects of our fear).

## An *Agency Via Awareness* Case Conceptualization

Below is an illustration of the Agency via Awareness framework applied to a specific fictionalized client with a relatively generalized deficit in agential functioning and corresponding awareness, along with the role that various therapeutic approaches and techniques might play as part of an Agency via Awareness treatment conceptualization.

Ronald is a 23-year-old bisexual man who works as a male escort. He was diagnosed with borderline personality disorder following a suicide attempt at age 21, but he has never previously worked with a therapist. He has a complex trauma history that includes childhood sexual abuse by his aunt and later by his older brother. He has a turbulent, on-and-off relationship with Joe, an ex-client, with whom he frequently gets into verbal and physical fights in which Ronald gets extremely upset for no obvious reason. At times these rage episodes lead him to hit Joe and threaten to kill himself. He has been legally mandated to see a therapist following an incident in which he went to Joe's house at night and scratched his car while Joe was home and asleep. According to Ronald, this action was triggered by Joe's brief mention of a previous boyfriend. Ronald also describes extremely mixed feelings about his work as an escort, where he is exposed to sexual abuse by his boss and mistreatment by some of his clients. He describes these “occupational hazards” as part of his job, but also speaks of wishing for a career that does not involve sex work.

In what respects does Ronald currently lack agency over his life? Besides the external threat of incarceration now facing him, he is caught in a cycle of intensely distressing emotions and impulsive, self-sabotaging behaviors that prevent him from experiencing the satisfaction or intimacy he seeks from his work or relationships. This pattern, in turn, likely reflects an implicit representation of himself as deeply helpless and of his relationships as unstable and unpredictable, formed through repeated early experiences of being abused and abandoned by those close to him. Ronald is largely unaware that this is how he implicitly views himself and the world, likely due to a mix of cognitive and motivational factors: e.g., he would not have had the cognitive tools to contextualize and make sense of his painful experiences as a child, and he has likely defended against full awareness of the grim and terrifying view that got formed as a result. As such, he has not been able to do the agential work of reflecting on his representation and updating it in light of his adult reality, which includes access to a much wider variety of relational experiences, action affordances, and cognitive tools than he had available as a child. Thus, for example, he still experiences any perceived signs of rejection or criticism through the filter of his implicit representations, such that they trigger intense and threatening emotions (like self-hatred, hopelessness, and fear of abandonment) against which he reflexively defends (e.g., through explosive rage at Joe). These defensive reactions, in turn, preclude him from attending to or reflecting on more nuanced aspects of the situation (e.g., Joe's subtle efforts to reconnect even while setting boundaries) that might otherwise help update his perspective and moderate his response.

Let us consider how we might draw upon existing therapeutic approaches to foster Ronald's agency by increasing his awareness. First we might focus on building an attuned and empathic therapeutic alliance with him to provide the experience of a different kind of relationship than he has previously encountered: one where it is safe and rewarding to be vulnerable with another person, and where he can be accepted and cared for even after revealing his supposedly “damaged” parts. This new relational awareness might then increase his willingness to explore and attend to previously unadmitted aspects of his emotional experience, thus setting the stage for the further psychodynamic or emotion-focused work of helping Ronald acknowledge the underlying hurt and fear of abandonment that lead him to behave in aggressive, self-sabotaging ways (as when he heard Joe mention his ex-boyfriend). Transferential work could further provide insight into Ronald's typical relational patterns and improve his reflective functioning—i.e., his nuanced awareness of how he is in relationships and how he and others may think and feel in response to his actions.

Once Ronald has fuller awareness of the emotions driving his aggressive behavior, he is better positioned to reflect on where these emotions and behavior patterns originated. For instance, we might utilize a trauma-focused psychodynamic, cognitive, or behavioral approach to help him process (albeit by different methods) the memories, emotions, and representations associated with his childhood abuse, thus gaining awareness of the connections between these past experiences and his current distrust in others as well as his career choice. Moreover, through a CBT approach such as cognitive processing therapy (CPT; Resick et al., 2017), Ronald may become aware of and check his trauma-induced beliefs about himself and others—e.g., that he is unlovable, that others cannot be trusted—against the evidence of external reality. For instance, he might be guided to attend to and reflect on any cognitive distortions that maintain these beliefs (such as overgeneralizing from “I was unable to stop the abuse” to “I am incapable of controlling anything that happens to me”), and to explore and integrate contrary evidence (e.g., that confiding in friends does not always scare them off, and sometimes actually builds closeness) into his growing awareness of himself, other people, and the world.

Before Ronald can engage in the kind of corrective experiences described above, he may need to become aware of how his distressing emotions are triggered and learn how to tolerate or down-regulate them in the moment. Toward this end, we might help him learn adaptive coping skills from CBT or DBT, such as mindfulness or sensory awareness strategies (Linehan, 2015), that ground him in the immediate awareness that he is safe and that his emotional pain will pass. By utilizing such skills, he would also gain awareness about his inner processes from the moment of being triggered to getting upset and acting out, and also about ways to influence these processes, thus further increasing his agency.

Once Ronald has updated and deepened his awareness of himself and the kinds of relationships and agential pursuits possible to him, he may further benefit from ACT work focused on articulating the values and goals he particularly wants to pursue, and translating these values and goals into action steps. For instance, upon reflection, he may want to work on mending his relationship with Joe, or he may choose to pursue a different relationship. Likewise, once he has internalized his awareness of his own efficacy enough to consider leaving his job as an escort, he may need help reflecting on what he values in a workplace as he selects new career paths to explore. By acting on these reflections through ongoing agential work, he would further clarify his values and accrue new experiences that further deepen his awareness of the more stable and authentic forms of human connection and goal-pursuit that are possible to him. Thus, he would gain agency by exploring, attending to, reflecting on, and applying his new awareness of various relevant aspects of himself and his world.

## Clinical Utility of Agency Via Awareness Framework

As the above example illustrates, some psychotherapy approaches emphasize increasing awareness of internal experience, as in the case of psychodynamic therapy; others put more emphasis on awareness of the external world, as in the case of CBT; and still others put more focus on increasing the exercise of mental and/or physical agency, as in the case of ACT and DBT. Despite these different emphases, we posit that each approach addresses aspects of the same underlying Agency via Awareness meta-process. Crucially, however, by adopting our proposed framework as an explicit guide to clinical decision-making, therapists would gain a more balanced and integrated view of these specific processes and the respective roles they play in therapeutic change. The fundamental question that guides each decision would be, “What further awareness does this client need in order to increase their agency over the aspects of life they are struggling with, and how can I help them gain it?” The answer to this question, in turn, would set the standard for determining when and how to facilitate a given therapeutic process within the context of a given client and situation. For instance, what counts as a “problem” to be addressed in therapy would depend on where the client is experiencing psychological constraints upon their agency, and what experiences they need to encounter, attend to, reflect on, or apply in order for these constraints to be removed. Of note, the clinician's assessment of this may at times differ from the client's: for instance, Ronald in our example may initially report that his biggest problem is that “Joe keeps jerking me around,” and that his goal for therapy is to “get him off my back.” After some initial assessment, however, the therapist may conclude that Ronald's difficulties with Joe are symptomatic of a broader, deeper pattern of expecting and thus inadvertently inviting rejection and abandonment in various aspects of his life and relationships. Likewise, in determining what corrective experiences a client needs and when, the clinician would be guided by an assessment of the awareness they need to acquire, access, or further integrate and internalize. For instance, Ronald may need to become aware that he has a secure emotional base to turn to before he will even consider the possibility that his behavior may be contributing to his difficulties. In that case, he may first need to experience a warm, caring therapeutic relationship that survives and works through several alliance ruptures (Eubanks et al., 2018) before honestly grappling with the question of why he really became an escort or why he behaves aggressively toward Joe. By contrast, a different client may already have some awareness upon coming into therapy that they are capable of loving and being loved, but may be afraid to break up with their long-time partner and seek a more satisfying relationship. In that case, the client may need the experience of actually enjoying some time alone without their partner, or of formulating a break-up plan and role-playing some assertive communication strategies. Of note, conceptualizing the case in these terms also informs the nature and style of the therapeutic relationship, which might be more focused on the transference-countertransference and other here-and-now aspects of the relationship in one case, vs. taking a more directive form that centers around collaborative tasks and awareness-building exercises in another case.

In this way, the Agency via Awareness framework can provide us with a parsimonious understanding of and vocabulary for the many stages and manifestations of therapeutic change. Armed with a conceptual framework for understanding therapy's core aim (the *why*) and meta-process (the *how*), therapists can make more informed, client-tailored decisions at every stage of the therapeutic process. Rather than falling into a rote routine of providing certain interventions for certain conditions, a clinician can make a moment-by-moment determination of what kind of awareness-building is needed in order to increase the client's agency. For instance, if a client has not developed the mental agency to disengage from their thoughts and reflect on them, it may be important to provide some self-reflective, mindful awareness or metacognitive strategies before (or in addition to) providing any particular content knowledge or interpretations: otherwise the therapist may risk facing resistance or pushing out one set of passively accepted convictions to make room for another, without having empowered the client to engage in their own agential work of building awareness. If a client already exhibits a sufficient degree of mental agency, but lacks specific awareness about the likelihood that certain feared outcomes will occur, it may be more appropriate to provide psychoeducation or present the relevant counterevidence, as the client will likely be able to weigh it and reach an informed conclusion independently of the therapist. Still another client may have a generally high level of mental agency but may not be exercising it with respect to a particularly painful aspect of their experience, such as a traumatic memory. In such a case, the therapist would accomplish little by repeatedly presenting the client with the knowledge they are avoiding (e.g., that a deceased loved one is never coming back). Rather, the therapist's task is to inspire and empower the client's own agential decision to attend to this knowledge, such as by providing a safe and empathic holding environment for the client's grief, or with a gentle reminder that avoiding reality only tends to make things worse (Linehan, 2015). Thus, by adopting the Agency via Awareness framework, clinicians can learn and capitalize on the distinctive strengths of each therapeutic approach, while keeping in mind the fundamental aim and meta-process that unifies them all.

## Discussion

The psychotherapy field suffers from a serious and persistent lack of theoretical unity (see Goldfried, 2019). Common factors researchers have worked to overcome this problem by identifying processes and principles that lead to therapeutic change across treatment modalities. This approach offers well-documented advantages over the traditional approach of pitting broad, entrenched theoretical orientations against each other. For instance, it facilitates common understanding and idea exchange among researchers and practitioners of different theoretical persuasions, and it enhances the quality and consistency of clinical training by offering a parsimonious set of empirically-validated principles that trainees can flexibly apply regardless of their orientation. As we have argued here, however, the common factors approach is limited by its agnosticism regarding the theoretical assumptions that underlie and conceptually unite these common factors. Whether or not they are aware of it, therapists inevitably operate on certain theoretical assumptions about the fundamental aims and mechanisms of psychotherapy. Our goal here is to make explicit the theoretical assumptions that implicitly underlie a wide range of established therapeutic principles and practices, thus affording clinicians the ability to reflect critically on these assumptions, and to apply them more systematically and judiciously to wide-ranging clinical situations. Putting this in the terms of our proposed framework: by gaining awareness of their implicit therapeutic assumptions, therapists will gain increased agency over whether, when, and how they apply those assumptions in therapy.

Of note, our goal here is not to arbitrate between the existing theoretical orientations within our field. Rather, it is to look afresh at the processes, principles, and techniques known to characterize a range of effective therapies, and to articulate the common theoretical assumptions that are implicit in all or most of them. Our framework identifies core theoretical assumptions behind a broad range of common therapeutic approaches, with the aim of providing a simple framework for understanding and informing the psychotherapy process. We have argued that the vast majority of therapy approaches share a common theoretical underpinning, in that they aim to increase client agency by facilitating increased awareness of the aspects of internal and/or external reality that are relevant to guiding and motivating their (mental or physical) actions. Therapists may do this by presenting clients with new experiences, encouraging attention and reflection on past and/or current experiences, or fostering the application of the client's resulting awareness to the pursuit and exploration of further new experiences. We have also demonstrated that each therapy approach stresses different aspects of awareness as relevant in order to increase client agency, that different therapy orientations put more emphasis on the agential rather than the awareness aspect and vice versa, and finally, that the work of building awareness is itself an inherently agential process.

One advantage of explicitly conceptualizing therapy in terms of its promotion of Agency via Awareness is that it would resolve a number of controversies and provide a common conceptual foundation for a number of seemingly disparate theoretical frameworks within the field. To take one example, consider the current conceptual standoff between “process-focused” and more “content-focused” therapies (see Harley, 2015). Interestingly, therapeutic approaches rooted in the Psychoanalytic and Behaviorist traditions have largely converged in their prioritization of process, albeit in different ways and for somewhat different reasons. The Psychoanalytic tradition explicitly views conscious thought content as being at the mercy of reality-distorting motives and defenses that operate outside of awareness. As such, any effort to engage directly with this content—such as by offering counter-evidence or checking its logical validity—would just perpetuate the illusion of objectivity created by the patient's defensive processes, instead of bringing those processes to light. Meanwhile the Behaviorist tradition explicitly regards thoughts as conditioned behaviors controlled by environmental contingencies, leaving no theoretical room for checking their veracity or modifying them based on evidence (e.g., Gross and Fox, 2009). Both traditions thus emphasize the importance of changing the process by which patients relate to their own thoughts: how attached they are to those thoughts, how flexibly they are able to move between or away from them, and so on. One might say that these traditions emphasize agency (and, in some cases, awareness of internal reality) while deemphasizing awareness of external reality.

By contrast, traditional cognitive approaches rooted in “information processing” models of human cognition tend to focus on aligning patients' thoughts and beliefs with external reality (e.g., via cognitive restructuring and behavioral experiments), but are less attentive to the different motivations that might energize a person's agential thought processes. As such, a cognitive psychologist might err on the side of trying, in effect, to force awareness of reality on someone who defensively resists it, without first identifying or addressing the source of that resistance. The tendency to view people as information processing machines, in other words, may lead to an emphasis on the content of awareness, but an underemphasis on the mental agency required to choose whether and how to engage with such content to begin with.

If we adopt the Agency via Awareness model proposed here, the apparent conflict between content- and process-focused approaches would dissolve. As understood through our Agency via Awareness framework, each of these emphases complements and depends on the other, and both have a place in the meta-process of enhancing agency through increasing awareness. Specifically, we would come to understand the pursuit of internalized awareness—i.e., accurate, accessible, personally meaningful content—as itself an agential metacognitive *process* motivated by the desire to enhance one's agential functioning. Clinical decisions about what aspects of this meta-process to emphasize at any given time would depend on where a given client is stuck, and what form of inspiration, experience, suggestion, or direction will help them get unstuck.

Likewise, our model provides a theoretical context for understanding and appropriately applying a range of findings from the systematic treatment selection literature (Beutler and Clarkin, 1990). For instance, a meta-analysis by Beutler et al. (2018) found that patients higher in “reactance” (i.e., strong resistance to the therapist's interventions) benefit more from exploratory, non-directive approaches, whereas less reactant patients respond better to structured, directive approaches. Interestingly, Beutler and colleagues attribute these findings to the “fear of losing some aspect of personal freedom” (Beutler et al., 2018, p. 1953) that reactant patients experience when a therapist directs them to behave in a specific way. Understood through our framework, these patients likely experience a threat to their agency when directed to act in ways that they have not come to endorse by their own lights, i.e., through their own agential process of building awareness. Of note, our framework would highlight the further need to distinguish between “low reactance” patients who actively comply with the therapist's guidance because they already have an internalized, independently held awareness of their reasons for seeking treatment, vs. those who are pressured to comply because they struggle with the mental agency needed to check or question the therapist's authority. The latter patients may also appear to benefit more from directive treatment than the more reactant patients, at least in the short term, but research suggests that this kind of passive, externally motivated compliance does not produce robust or enduring treatment gains (e.g., Orlinsky et al., 2004; Tryon and Winograd, 2011; see Ryan et al., 2011, for a review). By conceptualizing a given patient's therapeutic needs in terms of our framework, therapists would be able to tailor treatment not only based on whether a client is showing high levels of resistance, but also the level of a client's agential functioning and the kind of exploration or direction that would thus be most helpful in enhancing it.

### Cultural Considerations

Psychotherapy inherently focuses on internal processes of the patient, and has been criticized for often disregarding external, cultural or societal variations and restrictions (Sue et al., 2019). Cultural differences are especially salient with respect to an individual's agency, where societal and cultural factors often pose limitations. Marginalized groups within a society have limited agency compared to dominant groups (Sue, 2003), and psychotherapy research and practice are often biased in ascribing the same level of agency to these clients (e.g., Fors, 2018, Goodman and Gorski, 2015). For example, the barrier of wage discrimination may limit women's resources to develop or realize agentic plans for their own development (Bart et al., 2019). These concerns highlight the importance of considering the role of cultural and societal constraints when assessing a patient's baseline level of agential functioning, but do not in themselves preclude working to increase the patient's agency over the course of treatment; indeed, awareness of the relevant cultural and societal constraints may enable the therapist to intervene more effectively on the patient's agential functioning, such as by helping the client become aware of these cultural limitations and also the agency they have over how they relate to, question, confront, or accept these limitations (Fors, 2018).

Furthermore, as critical discourse analysis argues, the negotiation of client agency in therapy toward a more agential position, that is, being in control and having choices, may not be universal but represent a contemporary Western cultural ideal, which promotes self-contained individualism (Sampson, 1989), self-determination, and clear boundaries with others (Madill and Doherty, 1994), and often regards submission to others' wishes as problematic. Individuals with cultural backgrounds that promote more collectivistic values may face discrepancies between cultural norms and ideals regarding individual agency (Zane and Song, 2007). While resolving the philosophical and sociopolitical complexities involved in this debate is admittedly beyond the scope of this manuscript, therapists can still facilitate the recognition of these various cultural contexts and the values and expectations inherent in them, and encourage their clients to become aware of their culturally embedded values and take a reflected, agential approach regarding the extent to which they accept or reject them. Accepting one's cultural values, even if they promote interpersonal harmony over self-assertiveness, may be just as agential an act as any other, when it is preceded by a process of self-reflection, learning, and internalized awareness of one's relatedness to one's community and its values (La Roche, 2005).

### Limitations and Future Directions

Although we have attempted to illustrate how the Agency via Awareness meta-process manifests across wide-ranging orientations and clinical situations, we recognize that there are vastly more treatment approaches, formats, and client types than we have been able to cover here. Future research will be needed to explore the generalizability of this framework to other therapeutic modalities and clinical contexts. More generally, empirical validation of our hypothesized Agency via Awareness meta-process in psychotherapy is needed. Given that our conceptualization of agency and awareness, respectively, encompasses many specific facets and dimensions of these constructs that have been operationalized and measured in prior research, these established measures can be combined and triangulated in the context of psychotherapy to test various aspects of our hypothesized meta-process. For instance, we would hypothesize that patients' agency within the therapy context, as measured by the Therapeutic Agency Inventory (Huber et al., 2019), would be related to and preceded by their observer-rated insight (Jennissen et al., 2018), emotional awareness (Høglend and Hagtvet, 2019), and reflective functioning levels (Fonagy and Target, 1996; Fonagy et al., 2016), as well as less frequent use of immature defenses (Perry, 1990). Likewise, we would hypothesize that increases in clients' awareness (Geurtzen et al., 2020) and internalization (Pelletier et al., 1997) of their own therapeutic goals would precede and predict increases in therapeutic gains as well as general self-efficacy (Schwarzer and Jerusalem, 1995)—a measure closely tied to our understanding of physical agency.

Given our understanding of awareness and agency as inextricably related aspects of this meta-process, we are also currently developing new observer-rated measures that assess the interplay between these variables over the course of therapy. These measures aim to capture the content as well as the depth and accessibility of the client's awareness across various life domains, as well as the client's engagement in the agential processes of exploring, attending to, reflecting on, and applying new or existing knowledge. For instance, we aim to assess the extent to which clients passively “take in” or appear to mull over the claims and recommendations provided by the therapist, as attested by the questions they ask, the ways they relate the therapist's guidance to aspects of their own experience, etc. We will then evaluate whether these within-session markers of mental agency and awareness relate to symptom severity and predict improvement between sessions and over the course of the treatment.

Once validated, these measures will allow for research directly examining whether the Agency via Awareness meta-process represents a common mechanism of change across therapy modalities. For instance, studies could: (1) establish when, how, and to what extent different therapists and modalities focus on increasing awareness, increasing agency, or both; (2) measure the mediating and moderating effects of these processes and their combination on symptom improvement and therapy outcome, (3) identify the differential effects of interventions emphasizing awareness vs. agency in various conditions, (4) explore whether interventions focusing on different aspects of the meta-process are more beneficial for different individuals and/or at different phases of therapy, and (5) establish whether therapists' facilitation of awareness and/or agential work results in a better therapeutic relationship. Finally, this framework can guide the development and testing of future integrative therapeutic approaches that explicitly incorporate the Agency via Awareness language and conceptual model.

## Conclusion

Pending these validation efforts, our Agency via Awareness framework stands to offer an explicit theoretical model that summarizes what wide-ranging effective therapies *actually do* and *why they do it*, thus empowering clinicians to do their work more flexibly and deliberately. Defending the need for an agential theory of human psychology to guide practical life and treatment, Bandura (1998) wrote: “The value of a psychological theory is judged by three criteria. It must have explanatory power, it must have predictive power, and, in the final analysis, it must demonstrate operative power to improve the human condition” (p. 26). Such a theory, he wrote, would recognize that “people are agents of experiences rather than simply undergoers of experiences. The sensory, motor, and cerebral systems are tools that people use to accomplish the tasks and goals that give meaning, direction, and satisfaction to their lives” (p. 5). We propose that it is due time for the field of psychotherapy to align around such a theory, embracing the assumptions that are already inherent in the therapeutic enterprise.

## Data Availability Statement

The original contributions presented in the study are included in the article/supplementary material, further inquiries can be directed to the corresponding author/s.

## Author Contributions

EG and VB both contributed to the conception and writing up the study. All authors contributed to the article and approved the submitted version.

## Conflict of Interest

The authors declare that the research was conducted in the absence of any commercial or financial relationships that could be construed as a potential conflict of interest.
